# Phylogeny reconstruction based on the length distribution of *k*-mismatch common substrings

**DOI:** 10.1186/s13015-017-0118-8

**Published:** 2017-12-11

**Authors:** Burkhard Morgenstern, Svenja Schöbel, Chris-André Leimeister

**Affiliations:** Department of Bioinformatics, Institute of Microbiology and Genetics, University of Goettingen, Goldschmidtstr. 1, 37077 Göttingen, Germany

**Keywords:** Alignment-free, Phylogeny, Kmacs, Average common substring, Pattern matching

## Abstract

**Background:**

Various approaches to alignment-free sequence comparison are based on the length of exact or inexact word matches between pairs of input sequences. Haubold et al. (J Comput Biol 16:1487–1500, 2009) showed how the average number of substitutions per position between two DNA sequences can be estimated based on the average length of exact common substrings.

**Results:**

In this paper, we study the length distribution of *k*-mismatch common substrings between two sequences. We show that the number of substitutions per position can be accurately estimated from the position of a local maximum in the length distribution of their *k*-mismatch common substrings.

## Background

Phylogenetic distances between DNA or protein sequences are usually estimated based on pairwise or multiple sequence alignments. Since sequence alignment is computationally expensive, alignment-free phylogeny approaches have become popular in recent years, see Vinga [1] for a review. Some of these approaches compare the word composition [2–5] or spaced-word composition [6–9] of sequences using a fixed word length or patterns of match and don’t-care positions, respectively. Other approaches are based on the *matching statistics* [10], that is on the length of common substrings of the input sequences [11, 12]. All these methods are much faster than traditional alignment-based approaches. A disadvantage of most word-based approaches to phylogeny reconstruction is that they are not based on explicit models of molecular evolution. Instead of estimating distances in a statistically rigorous way, they only return rough measures of sequence similarity or dissimilarity.

The *average common substring (ACS)* approach [11] calculates for each position in one sequence the length of the longest substring starting at this position that matches a substring of the other sequence. The average length of these substring matches is then used to quantify the similarity between two sequences based on information-theoretical considerations; these similarity values are finally transformed into symmetric distance values. More recently, we generalized this approach by using common substrings with *k* mismatches instead of exact substring matches [13]. To assign distance values to sequence pairs, we used the same information-theoretical approach that is used in *ACS*. Since there is no exact solution to the *k-mismatch longest common substring problem* that is fast enough to be applied to long genomic sequences, we proposed a simple heuristic: we first search for longest *exact* matches and then extend these matches until the* k* + 1st mismatch occurs. Distances are then calculated from the *average* length of these *k*-mismatch common substrings similarly as in *ACS*; the implementation of this approach is called *kmacs*. Various algorithms have been proposed in recent years to calculate exact or approximate solutions for the *k-mismatch average common substring problem* and have been applied to phylogeny reconstruction [14–20]. Like *ACS* and *kmacs*, these approaches are not based on stochastic models.

To our knowledge, the first alignment-free approach to estimate the phylogenetic distance between two DNA sequences in a statistically rigorous way was the program *kr* by Haubold et al. [21]. These authors showed that the average number of nucleotide substitutions per position between two DNA sequences can be estimated by calculating for each position *i* in one sequence the length of the shortest substring starting at *i* that does not occur in the other sequence, see also [22, 23]. This way, phylogenetic distances between DNA sequences can be accurately estimated for up to around 0.5 substitutions per position. Some other, more recent, alignment-free approaches also estimate phylogenetic distances based on stochastic models of molecular evolution, namely *Co-phylog* [24], *andi* [25], an approach based on the *number* of (spaced) word matches [7] and *Filtered Spaced Word Matches* [26].

In this paper, we propose an approach to estimate phylogenetic distances based on the length distribution of *k*-mismatch common substrings. The manuscript is organized as follows. In the next section, we introduce some notation and the stochastic model of sequence evolution that we are using. In the following two sections, we recapitulate a result from [21] on the length distribution of longest common substrings, we generalize this to *k*-mismatch longest common substrings, and we study the length distribution of *k*-mismatch common substrings returned by the *kmacs* heuristic [13]. Then, we introduce our new approach to estimate phylogenetic distances and explain some implementation details. In the final sections, we report on benchmarking results, discuss these results and address some possible future developments. We should mention that “*k*-mismatch longest common substrings” and “Heuristics used in kmacs” sections are not necessary to understand our new approach that is introduced in “Distance estimation” section. We added these two sections for completeness, and since they may be used for alternative ways of phylogenetic distance estimation. But readers who are mainly interested in our approach to distance estimation can skip these sections.

## Sequence model and notation

We use standard notation such as used in [27]. For a sequence *S* of length *L* over some alphabet, *S*(*i*) is the *i*th character in *S*. *S*[*i*..*j*] denotes the (contiguous) substring from *i* to *j*; we say that *S*[*i*..*j*] is a *substring at i*. In the following, we consider two DNA sequences $S_1$ and $S_2$ that are thought to have descended from an unknown common ancestor under the *Jukes-Cantor* model [28]. That is, we assume that substitutions at different positions are independent of each other, that we have a constant substitution rate at all positions and that all substitutions occur with the same probability. We therefore have a *match probability* *p* and a *background probability* *q* such that $P\left( S_1(i) = S_2(j)\right) = p$ if $S_1(i)$ and $S_2(j)$ descend from the same position in the hypothetical ancestral sequence—in which case $S_1(i)$ and $S_2(j)$ are called ‘homologue’—and $P\left( S_1(i) = S_2(j)\right) = q$ otherwise (‘background’).

Moreover, we use a gap-free model of evolution where $S_1$ and $S_2$ have the same length *L*, to simplify the considerations below. With this model, $S_1(i)$ and $S_2(j)$ are ‘homologue’ if and only if $i=j$, so we have

$$\begin{aligned} P\left( S_1(i) = S_2(j)\right) = \left\{ \begin{array}{ll} p \quad{} \text { if } i = j\\ q \quad {} \text { else } \\ \end{array} \right. \end{aligned}$$

Similarly, we call a pair of equal-length substrings of $S_1$ and $S_2$ *homologue* if they start at the same respective positions in $S_1$ and $S_2$, and *background* otherwise. The background match probability *q* can be easily estimated from the relative frequencies of the four nucleotides. The main goal of the present study is to estimate the probability *p*. The *distance* between $S_1$ and $S_2$, defined as the number of substitutions per position since two sequences diverged from their last common ancestor, can then be obtained from *p* by the usual *Jukes-Cantor* correction. Note that, with our gap-free model, it is trivial to estimate *p* as the relative frequency of positions *i* where $S_i(i)$ equals $S_2(i)$. However, we will apply our results to real-world sequences with insertions and deletions where such a trivial approach is not possible.

## *k*-mismatch longest common substrings

For positions *i* and *j* in sequence $S_1$ and $S_2$, respectively, we define random variables

$$\begin{aligned} X_{i,j} = \max \{l: S_1[i..i+l-1] = S_2[j..j+l-1] \} \end{aligned}$$

**Fig. 1:**
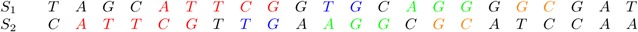
*k*-mismatch common substrings with $k=2$. For position $i=5$ in $S_1$, *kmacs* searches the longest substring of $S_1$ starting at *i* that exactly matches a substring of $S_2$. This is the substring starting at $i^*=2$ in $S_2$ (matching substrings shown in red). It then extends this match without gaps until the $k+1$st mismatch is reached. In this example, the *k-mismatch common substring* would consist of the red, blue and green substrings and has length 12. In the paper, the lengths of these *k*-mismatch common substrings are modelled by the random variables $X_i^{(k)}$, defined in (1). The original version of *kmacs* uses the average length of these *k*-mismatch common substrings to assign a distance value to a pair of sequences. In our *modified* implementation of *kmacs*, we consider the *k*-mismatch *extension* of the longest common substring at *i*. That is, the program would return the length of the *k*-mismatch substring match that starts *after* the first mismatch following the longest common substring. In our example, for $i=5,$ this would be the substring match starting with ‘*T*’ at position 11 in $S_1$ and at position 8 in $S_2$, consisting of the blue, green and orange matches; the length of this *k*-mismatch substring *extension* would be 9. The length of these *k*-mismatch *extensions* are modelled by the random variable $\hat{X}_i^{(k)},$ defined in (16)

That is, $X_{i,j}$ is the length of the longest substring at *i* that exactly matches a substring at *j*. Next, we define

1 $$\begin{aligned} X_i = \max _{1\le j\le L} X_{i,j} \end{aligned}$$

as the length of the *longest substring* at *i* that matches a substring of $S_2$ *anywhere* in the sequence, see Fig. 1 for an example.

In the following, we ignore edge effects, which is justified if long sequences are compared since the probability of *k*-mismatch common substrings of length *m* decreases rapidly if *m* increases. With this simplification, we have

2 $$\begin{aligned} P(X_{i,j} < n ) = 1 - P(X_{i,j} \ge n ) = \left\{ \begin{array}{ll} 1-p^n {} \quad \text { if } i = j \\ 1-q^n {} \quad \text { else } \\ \end{array} \right. \end{aligned}$$

If, in addition, we assume equilibrium frequencies for the nucleotides, i.e. if we assume that each nucleotide occurs at each sequence position with probability 0.25, the random variables $X_{i,j}$ and $X_{i',j'}$ are independent of each other whenever $j-i\not =j'-i'$ holds. In this case, we have for $n\le L-i+1$

3 $$\begin{aligned} P(X_{i} < n) & = P(X_{{i,1}} < n \wedge \ldots \wedge X_{{i,L}} < n) \\ & = P(X_{{i,1}} < n)\cdot \ldots \cdot P(X_{{i,L}} < n) \\ &= P(X_{{i,1}} < n)\cdot \ldots \cdot P(X_{{i,L - n + 1}} < n) \\ & = (1 - q^{n} )^{{L - n}} \cdot (1 - p^{n} ) \\ \end{aligned}$$

and

$$\begin{aligned} P(X_{i} = n) & = P(X_{i} < n + 1) - P(X_{i} {\text{ < }}n) \\ & \quad \quad = (1 - q^{{n + 1}} )^{{L - n - 1}} \cdot (1 - p^{{n + 1}} ) - (1 - q^{n} )^{{L - n}} \cdot (1 - p^{n} ) \\ \end{aligned}$$

so the expected length of the longest common substring at a given sequence position is

4 $$\begin{aligned} \sum _{n=1}^L n \cdot \left( (1-q^{n+1})^{{L-n-1}} \cdot (1-p^{n+1}) - \left(1-q^n \right)^{{L-n}} \cdot \left (1-p^n \right) \right) \end{aligned}$$

Next, we generalize the above considerations by looking at the average length of the *k*-*mismatch longest common substrings* between two sequences for some integer $k \ge 0$. That is, for a position *i* in one of the sequences, we consider the longest substring starting at *i* that matches some substring in the other sequence with a *Hamming distance* $\le k.$ Generalizing the above notation, we define random variables

$$\begin{aligned} X_{i,j}^{(k)} = \max \left\{ l: d_H\left( S_1[i..i+l-1],\;S_2[j..j+l-1]\right) {=\ } k \right\} \end{aligned}$$

where $d_H(\cdot ,\cdot )$ is the *Hamming distance* between two sequences. In other words, $X_{i,j}^{(k)}$ is the length of the longest substring starting at position *i* in sequence $S_1$ that matches a substring starting at position *j* in sequence $S_2$ with *k* mismatches. Accordingly, we define

$$\begin{aligned} X_{i}^{(k)} = \max _j X^{(k)}_{i,j} \end{aligned}$$

as the length of the longest *k*-mismatch substring at position *i*. As pointed out by Apostolico et al. [18], $X^{(k)}_{i,j}$ follows a *negative binomial distribution*, and we can write

5 $$\begin{aligned} P\left( X^{(k)}_{i,j} = n \right) = \left\{ \begin{array}{ll} {n \atopwithdelims ()k} p^{n-k} (1-p)^{k+1} {} \quad \text { if } i=j \\ {n \atopwithdelims ()k} q^{n-k} (1-q)^{k+1} {} \quad \text { else } \\ \end{array} \right. \end{aligned}$$

and

6 $$\begin{aligned} P\left( X^{(k)}_{i,j} \ge n \right) = \left\{ \begin{array}{ll} \sum _{k'\le k} {n \atopwithdelims ()k'} p^{n-k'} (1-p)^{k'} {} \quad \text { if } i=j \\ \sum _{k'\le k} {n \atopwithdelims ()k'} q^{n-k'} (1-q)^{k'} {} \quad \text { else } \\ \end{array} \right. \end{aligned}$$

Generalizing (3), we obtain for $n>k$

7 $$\begin{aligned} {P\left( X_{i}^{(k)} n\right) = }\nonumber \\\left( 1 - \sum _{k'\le k} {n \atopwithdelims ()k'} q^{n-k'} (1-q)^{k'}\right) ^{L} {-\;n}\cdot \left( 1 - \sum _{k'\le k} {n \atopwithdelims ()k'} p^{n-k'} (1-p)^{k'}\right) \end{aligned}$$

while we have

$$\begin{aligned} P\left( X_{i}^{(k)} < n\right) = \left\{ \begin{array}{ll} 1 {} \quad \text { if } n > L-i+1\\ 0 {} \quad \text { if } n \le k \\ \end{array} \right. \end{aligned}$$

Finally, we obtain

8 $$\begin{aligned} {P\left( X_i^{(k)}=n\right) = \left. \left( 1 - \sum _{k'\le k} {n+1 \atopwithdelims ()k'} q^{n+1-k'} (1-q)^{k'}\right) ^{L -{n-1}} \right. }\nonumber \\ \quad \qquad \quad \qquad \cdot \left( 1 - \sum _{k'\le k} {n+1\atopwithdelims ()k'} p^{n+1-k'} (1-p)^{k'}\right) \nonumber \\ \quad \qquad \quad \qquad - \left. \left( 1 - \sum _{k'\le k} {n \atopwithdelims ()k'} q^{n-k'} (1-q)^{k'}\right) ^{L} {-n} \cdot \left( 1 - \sum _{k'\le k} {n \atopwithdelims ()k'} p^{n-k'} (1-p)^{k'}\right) \right. \end{aligned}$$

from which one can obtain the expected length of the *k*-mismatch longest substrings.

## Heuristic used in *kmacs*

Since exact solutions for the *average k-mismatch common substring problem* are too time-consuming for large sequence sets, the program *kmacs* [13] uses a heuristic. In a first step, the program calculates for each position *i* in one sequence, the length of the longest substring starting at *i* that *exactly* matches a substring of the other sequence. *kmacs* then calculates the length of the longest gap-free *extension* of this exact match to the right-hand side with *k* mismatches. Using standard indexing structures, this can be done in $O (L\cdot k)$ time.

For sequences $S_1, S_2$ as above and a position *i* in $S_1$, let $j^*$ be a position in $S_2$ such that the $X_i$-length substring starting at *i* matches the $X_i$-length substring at $j^*$ in $S_2$. That is, the substring

$$\begin{aligned} S_2[j^*..j^* + X_i -1] \end{aligned}$$

is the longest substring of $S_2$ that matches a substring of $S_1$ at position *i*. In case there are several such positions in $S_2$, we assume for simplicity that $j^* \not = i$ holds (in the following, we only need to distinguish the cases $j^*=i$ and $j^*\not = i$, otherwise it does not matter how $j^*$ is chosen). Now, let the random variable $\tilde{X}^{(k)}_i$ be defined as the length of the *k*-mismatch common substring starting at *i* and $j^*$, so we have

9 $$\begin{aligned} \tilde{X}^{(k)}_i = X_{i,j^*}^{(k)} = X_i + X^{(k-1)}_{i+X_i,j^*+X_i} + 1 \end{aligned}$$

### **Theorem 1**

*For a pair of sequences as above,* $1 \le i \le L$ *and* $m\le {L - i + 1}$ *, the probability of the heuristic kmacs hit of having a length of m is given as*

$$\begin{aligned}&P\left( \tilde{X}^{(k)}_i = m\right) \\&= \ \ p^{m-k+1}(1-p)^{k+1} \sum _{m_1+m_2=m{-1}} (1 - q^{m_1+1})^{L-{m_1}} {m_2 \atopwithdelims ()k-1} \\& \quad + \sum _{m_1+m_2=m{-1}} \left[ (1-q^{m_1+1})^{L-{m_1}} - (1-q^{m_1})^{L-{m_1}} \right] \cdot (1-p^{m_1}) \\ & \quad \ \ \cdot {m_2 \atopwithdelims ()k-1} q^{m_2-k+1}(1-q)^k \\ \end{aligned}$$

### *Proof*

Distinguishing between ‘homologous’ and ‘background’ matches, and using the law of total probability, we can write

10 $$\begin{aligned} P\left( {\tilde{X}}^{(k)}_i = m\right) &=P\left( {\tilde{X}}^{(k)}_i = m \left| j^*=i\right) P(j^*=i)\right. \nonumber \\ & \quad+ P\left( \tilde{X}^{(k)}_i = m \left| j^*\not =i\right) P(j^*\not =i) \right. \end{aligned}$$

and with (5), we obtain

11 $$\begin{aligned}&P\left( \tilde{X}^{(k)}_i = m\left| j^*=i\right) \right. \nonumber \\&= \sum _{m_1+m_2=m{-1}} P(X_i = m_1 | j^*=i) P\left( X_{i+m_1{+1},i+m_1{+1}}^{(k-1)}=m_2\right) \nonumber \\&= \sum _{m_1+m_2=m{-1}} P(X_i = m_1 | j^*=i) {m_2 \atopwithdelims ()k-1} p^{m_2-k+1}(1-p)^k \end{aligned}$$

and

12 $$\begin{aligned} P(X_i = m_1 | j^*=i) &= \frac{P(X_{i,i}=m_1 \wedge j^*=i)}{P(j^*=i) } \nonumber \\& = \frac{P(X_{i,i}=m_1 \wedge X_{i,i} \ge X_{i,j}, j\not = i)}{P(j^*= i)} \nonumber \\& = \frac{P(X_{i,i}=m_1 \wedge X_{i,j}\le m_1, j\not = i)}{P(j^*= i)} \nonumber \\&= \frac{p^{m_1}(1-p) \cdot (1 - q^{m_1+1})^{L-{m_1}}}{P(j^*= i)} \end{aligned}$$

so with (11) and (12), the first summand in (10) becomes

13 $$\begin{aligned}& P\left( \tilde{X}^{(k)}_i = m\left| j^*=i\right) P(j^*=i) \right. \nonumber \\&= \sum _{m_1+m_2=m{-1}} P(X_i = m_1 | j^*=i) {m_2 \atopwithdelims ()k-1} p^{m_2-k+1}(1-p)^k \cdot P(j^*=i) \nonumber \\&= \sum _{m_1+m_2=m{-1}} \frac{p^{m_1}(1-p) \cdot (1 - q^{m_1+1})^{L-{m_1}}}{P(j^*= i)} \nonumber \\ & \quad \cdot \ \ {m_2 \atopwithdelims ()k-1} p^{m_2-k+1}(1-p)^k \cdot P(j^*=i) \nonumber \\&= \sum _{m_1+m_2=m{-1}} (1 - q^{m_1+1})^{L-{m_1}} {m_2 \atopwithdelims ()k-1} p^{m_1+m_2-k+1}(1-p)^{k+1} \nonumber \\&=\ \ p^{m-k+1}(1-p)^{k+1} \sum _{m_1+m_2=m{-1}} (1 - q^{m_1+1})^{L-{m_1}} {m_2 \atopwithdelims ()k-1} \end{aligned}$$

Similarly, for the second summand in (10), we note that

14 $$\begin{aligned}&P\left( \tilde{X}^{(k)}_i = m| j^*\not =i\right) \nonumber \\&= \sum _{m_1+m_2=m{-1}} P(X_i = m_1 | j^*\not =i) {m_2 \atopwithdelims ()k-1} q^{m_2-k+1}(1-q)^k \end{aligned}$$

and

15 $$\begin{aligned} P(X_i = m_1 | j^*\not =i) &= \frac{P(X_{i,j^*}=m_1 \wedge j^*\not =i)}{P(j^*\not =i) } \nonumber \\& = \frac{P(X_{i,j^*}=m_1 \wedge X_{i,i} < X_{i,j^*})}{P(j^*\not = i)} \nonumber \\ & = \frac{P(X_{i,j^*}=m_1 \wedge X_{i,i} < m_1 )}{P(j^*\not = i)} \nonumber \\ & = \frac{P(\max _{j\not =i}X_{i,j}=m_1 \wedge X_{i,i} < m_1 )}{P(j^*\not = i)} \nonumber \\ & = \frac{P(\max _{j\not =i}X_{i,j}=m_1) \cdot P( X_{i,i} < m_1 )}{P(j^*\not = i)}\nonumber \\ & = \frac{P(\max _{j\not =i}X_{i,j}=m_1)\cdot P( X_{i,i} < m_1)}{P(j^*\not = i)}\nonumber \\ & = \frac{\left[ (1-q^{m_1+1})^{L-{m_1}} - (1-q^{m_1})^{L-{m_1}} \right] \cdot (1-p^{m_1})}{P(j^*\not = i)} \end{aligned}$$

Thus, the second summand in (10) is given as

$$\begin{aligned}&P\left( \tilde{X}^{(k)}_i = m\left| j^*\not =i\right) P(j^*\not =i) \right. \\&= \sum _{m_1+m_2=m{-1}} P(X_i = m_1 | j^*\not =i) {m_2 \atopwithdelims ()k-1} q^{m_2-k+1}(1-q)^k \cdot P(j^*\not = i) \\&= \sum _{m_1+m_2=m{-1}} \frac{\left[ (1-q^{m_1+1})^{L-{m_1}} - (1-q^{m_1})^{L-{m_1}} \right] \cdot (1-p^{m_1})}{P(j^*\not = i)} \\&\ \ {m_2 \atopwithdelims ()k-1} q^{m_2-k+1}(1-q)^k \cdot P(j^*\not = i) \\&= \sum _{m_1+m_2=m{-1}} \left[ (1-q^{m_1+1})^{L-{m_1}} - (1-q^{m_1})^{L-{m_1}} \right] \cdot (1-p^{m_1}) \\&\ \ \cdot {m_2 \atopwithdelims ()k-1} q^{m_2-k+1}(1-q)^k \\ \end{aligned}$$

□

**Fig. 2:**
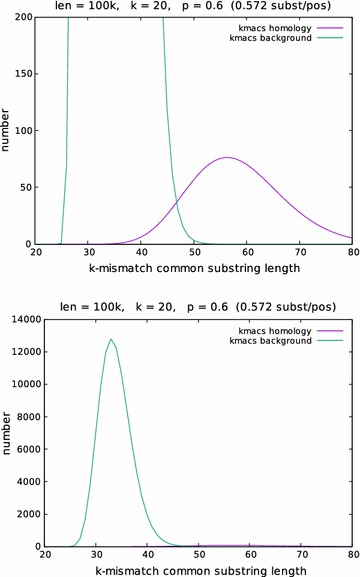
Theoretical length distribution of *k*-mismatch longest common substrings. The *expected* number of homologous and background *k*-mismatch longest common substrings of length *m*, returned by the *kmacs* heuristic, was calculated for $20 \le m \le 80$ using Theorem 1 for an indel-free pair of sequences of length $L=100$ kb, a match probability $p=0.6$ (corresponding to 0.57 substitutions per position) and $k=20$

For $1\le m \le L$, the expected number of *k*-mismatch common substrings of length *m* returned by the *kmacs* heuristics is given as $L \cdot P\left( \tilde{X}^{(k)}_i = m\right)$ and can be calculated using Theorem 1. Moreover, one can use the above considerations to calculate the length distributions of the *homologous* and *background* *k*-mismatch common substrings returned by *kmacs*. (Remember that, with our simple gap-free model, two substrings of $S_1$ and $S_2$, respectively, are called *homologous* if they start at the same positions and *background* otherwise.) The probabilities on the right-hand side of Eq. (10) can be used to calculate the *expected* number of *homologous* and *background* *k*-mismatch common substrings of length *m* returned by *kmacs*. In Fig. 2, these expected numbers are plotted against *m* for $L=100$ kb, $p=0.6$ and $k=20$.

## Distance estimation

Using Theorem 1, one could estimate the match probability *p*—and thereby the average number of substitutions per position—from the *empirical* average length of the *k*-mismatch common substrings returned by *kmacs* in a moment-based approach, similar to the approach proposed in [21].

A problem with this moment-based approach is that, for realistic values of *L* and *p*, one has $P(j^*=i) \ll P(j^*\not =i)$, so the above sum is heavily dominated by the ‘background’ part, i.e. by the second summand in (10). For the parameter values used in Fig. 2, for example, only 1% of the matches returned by* kmacs* represent homologies while 99% are background noise. There are, in principle, two ways to circumvent this problem. First, one could try to separate homologous from background matches using a suitable threshold value, similarly as we have done in our *Filtered Spaced Word Matches* approach [29]. But this is more difficult for *k*-mismatch common substrings, since there can be much more overlap between homologous and background matches than for *Spaced-Word* matches, see Fig. 2.

There is an alternative to this moment-based approach, however. As can be seen in Fig. 2, the length distribution of the *k*-mismatch longest common substrings is *bimodal*, with a first peak in the distribution corresponding to the background matches and the second peak corresponding to the homologous matches. We show that the number of substitutions per positions can be easily estimated from the position of this second peak.

**Fig. 3:**
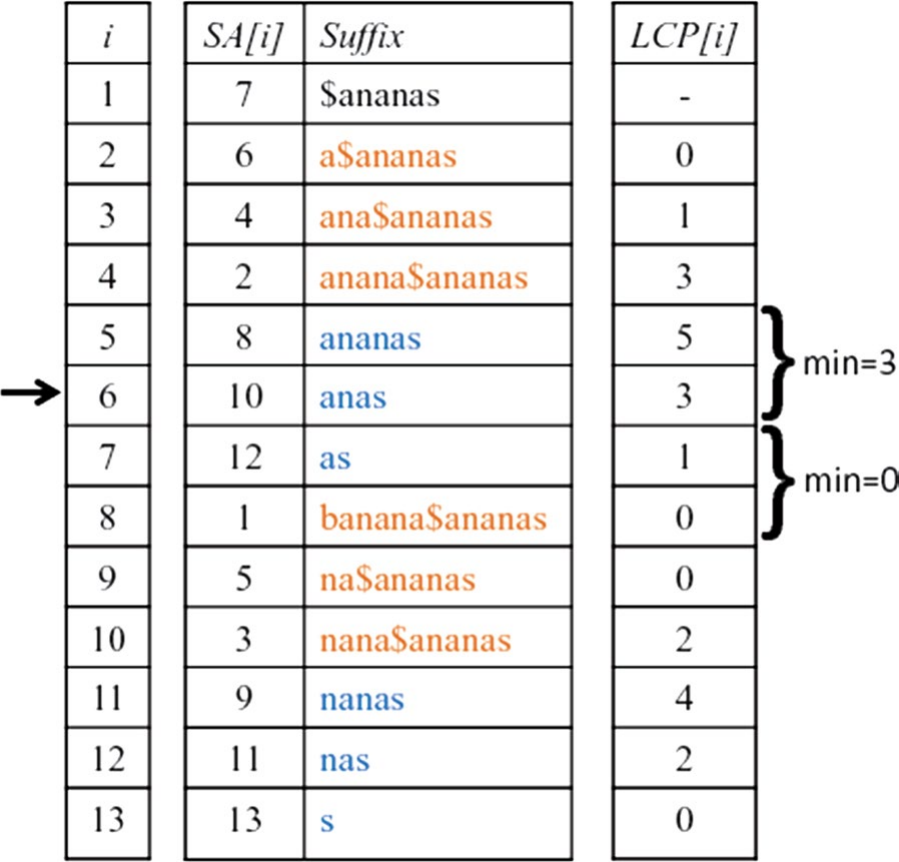
Enhanced suffix array. For sequences ‘banana’ and ‘ananas’, the *enhanced suffix array* is shown. Suffixes of the concatenated sequence are lexicographically ordered; a *longest common prefix (LCP)* array indicates the length of the longest common prefix of a suffix with its predecessor in the list (Figure taken from [13])

To simplify the following calculations, we ignore the longest exact match in Eq. (9), and consider only the length of the gap-free ‘extension’ of this match, see Fig. 1 for an illustration. To model the length of these *k*-mismatch *extensions*, we define define random variables

16 $$\begin{aligned} \hat{X}^{(k)}_i = \tilde{X}_{i}^{(k+1)} - X_i = X^{(k)}_{i+X_i+1,j^*+X_i+1} \end{aligned}$$

In other words, for a position *i* in sequence $S_1$, we are looking for the longest substring starting at *i* that exactly matches a substring of $S_2$. If $j^*$ is the starting position of this substring of $S_2$, we define $\hat{X}^{(k)}_i$ as the length of the longest possible substring of $S_1$ starting at position $i+ X_i + 1$ that matches a substring of $S_2$ starting at position $j^* + X_i + 1$ with a Hamming distance of *k*.

### **Theorem 2**

*Let* $\hat{X}^{(k)}_i$ *be defined as in (* 16 *). Then* $\hat{X}^{(k)}_i$ *is the sum of two unimodal distributions, a ‘homologous’ and a ‘background’ contribution, and the maximum of the ‘homologous’ contribution is reached at*

$$\begin{aligned} m_H = \left\lceil \frac{k}{1-p} -1 \right\rceil \end{aligned}$$

*and the maximum of the ‘background contribution’is reached at*

$$\begin{aligned} m_B = \left\lceil \frac{k}{1-q} -1 \right\rceil \end{aligned}$$

### *Proof*

As in (5), the distribution of $\hat{X}^{(k)}_i$ conditional on $j^*=i$ or $j^*\not =i$, respectively, can be easily calculated as

$$\begin{aligned} P\left( \hat{X}^{(k)}_i = m | j^*=i \right) = P\left( X^{(k)}_{i+ X_i+1,i+ X_i+1} = m \right) = {m \atopwithdelims ()k} p^{m-k} (1-p)^{k+1} \end{aligned}$$

and

$$\begin{aligned} P\left( \hat{X}^{(k)}_i = m\left| j^*\not = i \right) = {m \atopwithdelims ()k} q^{m-k} (1-q)^{k+1} \right. \end{aligned}$$

so we have

17 $$\begin{aligned} P\left( \hat{X}^{(k)}_i = m\right)&= P(j^* = i) {m \atopwithdelims ()k} p^{m-k} (1-p)^{k+1} \nonumber \\& \quad+ P(j^*\not = i) {m \atopwithdelims ()k} q^{m-k} (1-q)^{k+1} \end{aligned}$$

For the *homologous* part

$$\begin{aligned} H_k(m) = {P(j^*= i)} {m \atopwithdelims ()k} p^{m-k} (1-p)^{k+1} \end{aligned}$$

we obtain the recursion

$$\begin{aligned} H_k(m+1)= { \frac{m+1}{m+1-k}\cdot p \cdot H_k(m) } \end{aligned}$$

so we have $H_k(m) \le H_k(m+1)$ if and only if

18 $$\begin{aligned} \frac{ m+1-k }{m+1} \le p \end{aligned}$$

Similarly, the ‘background contribution’

$$\begin{aligned} B_k(m) = P(j^*\not = i) {m \atopwithdelims ()k} q^{m-k} (1-q)^{k+1} \end{aligned}$$

is increasing until

$$\begin{aligned} \frac{ m+1-k }{m+1} \le q \end{aligned}$$

holds, which concludes the proof of the theorem. □

The proof of Theorem 2 gives us lower and upper bounds for *p* and an easy approach to estimate *p* from the empirical length distribution of the *k*-mismatch extensions calculated by *kmacs*. Let $m_{\max }$ be the maximum of the *homologous* part of the distribution $\hat{X}^{(k)}_i$, i.e. we define

$$\begin{aligned} m_{\max } =\mathop{\text{argmax}}_m {m \atopwithdelims ()k} p^{m-k} (1-p)^{k+1} \end{aligned}$$

Then, by inserting $m_{\max }-1$ and $m_{\max }$ into inequality (18), we obtain

$$\begin{aligned} \frac{m_{\max }-k}{m_{\max }} \le p \le \frac{ m_{\max }+1-k }{m_{\max }+1} \end{aligned}$$

Finally, we use (18) to estimate *p* from the second maximum $m_E$ of the empirical distribution of $\hat{X}_i$ as

19 $$\begin{aligned} \hat{p} \approx \frac{ m_E +1-k }{m_E+1} \end{aligned}$$

For completeness, we calculate the probability $P(j^* = i)$. First we note that, by definition, for all *i*, we have

$$\begin{aligned} P(j^* = i) = P\left( X_{i,j}\,< \,X_{i,i} \quad \text { for all } j\not =i\right) \end{aligned}$$

so with the law of total probability and Eq. (2), we obtain

20 $$\begin{aligned} P(j^* = i) & = P\left( X_{i,j} < X_{i,i} \quad \text { for all } j\not =i\right) \nonumber \\ &= \sum _m P\left( X_{i,j} < X_{i,i} \quad \text { for all } j\not =i| X_{i,i} = m \right) P( X_{i,i} = m) \nonumber \\ & = \sum _m P\left( X_{i,j} < m \quad \text { for all } j\not =i \right) P( X_{i,i} = m) \nonumber \\ & = \sum _m \prod _{j\not =i}\, P( X_{i,j} < m)\, P( X_{i,i} = m) \nonumber \\ & = \sum _m (1-q^m)^{L -1} p^m (1-p) \end{aligned}$$

## Implementation

**Fig. 4:**
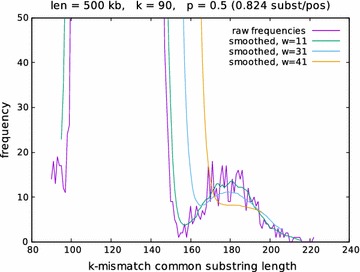
Empirical length distribution of *k*-mismatch common substring extensions. The number of *k*-mismatch extensions of length *m* was calculated with *kmacs* for a pair of simulated DNA sequences of length $L=500$ kb with $k=90$ and $80 \le m \le 240$. The plot shows the raw frequencies and smoothed distribution with different values for for the width *w* of the smoothing window. The hight of the ‘homologous’ peak is > 50,000

For each position *i* in one of the two input sequences, *kmacs* first searches the longest substring starting at *i* that exactly matches a substring of the other sequence. For a user-defined parameter *k*, the program then calculates the length of the longest possible gap-free extension with *k* mismatches of this exact hit. The original version of the program uses the average length of these *k*-mismatch common substrings (the initial exact match plus the $k-1$-mismatch extension after the first mismatch) to assign a distance value to a pair of sequences. We modified *kmacs* to output the length of the *extensions* of the identified matches only, ignoring these initial exact matches. Thus, to find *k*-mismatch common substrings, we ran *kmacs* with parameter $k+1$, and we consider the length of the *k*-mismatch extension *after* the first mismatch. For each possible length *m*, the modified program outputs the number *N*(*m*) of *k*-mismatch extensions of length *m*, starting after the first mismatch after the respective longest exact match.

To find for each position *i* in one sequence the length of the longest string at *i* matching a substring of the other sequences, *kmacs* uses a standard procedure based on *enhanced suffix arrays* [30], see Fig. 3. The algorithm first identifies the corresponding position in the suffix array. It then goes in both directions, up and down, in the suffix array until the first entry from the respective other sequence is found. In both cases, the minimum of the *LCP* values is recorded. The maximum of these two minima is the length of the longest substring in the other sequence matching a substring starting at *i*. In Fig. 3, for example, if *i* is position 3 in the string ananas, i.e. the 10th position in the concatenate string, the minimum *LCP* value until the first entry from banana is found, is 3 if one goes up the array and 0 if one goes down. Thus, the longest string in banana matching a substring starting at position 3 in ananas has length 3.

Note that, for a position *i* in one sequence, it is possible that there exists more than one maximal substring in the other sequence matching a substring at *i*. In this case, our modified algorithm uses *all* of these maximal substring matches, i.e. all maximal exact string matches are extended as described above. All these hits can be easily found in the suffix array by extending the search in upwards or downwards direction until the minimum of the *LCP* entries decreases. In the above example, there is a second occurrence of ana in banana which is found by moving one more position upwards (the corresponding *LCP* value is still 3).

In addition, we modified the original *kmacs* to ensure that, for each pair $(i',j')$ of positions from the two input sequences, the extended *k*-mismatch common substring starting at $(i',j')$ is counted only once. This is necessary for the following reason: if $S_1$ and $S_2$ share a long common substring *S*, then there will be many positions *i* in $S_1$ within *S* such that $j^*$ is at the corresponding position of *S* in $S_2$. In Fig. 1, for example, the red substring starting at positions 5 and 2, respectively, would be such a string *S*. Here, there are three positions *i* in $S_1$—positions 5, 6 and 7—such that the respective $j^*$ would be at the corresponding positions in $S_1$—at positions 2, 3 and 4, in this case. As a consequence, *all* maximal exact matches starting at these positions end before the first mismatch after the red substring—at positions 10 and 7—, so the *k*-mismatch *extensions* of *all* these exact matches start at positions $i'=11$ and $j'=8$ in $S_1$ and $S_2$, respectively. If *all* *k*-mismatches returned by *kmacs* would be counted, the extension starting after the red exact substring match would be counted three times. In real-world genomic sequences, such situations are common. Without the above correction, we observed isolated values *m* in the length distribution of the *k*-mismatch extensions, such that the number *N*(*m*) of *k*-mismatch extensions of length *m* is very high, while $N(m')$ is zero for neighbouring values $m'$.

**Fig. 5:**
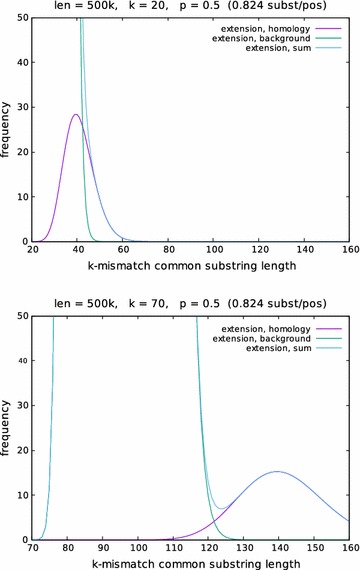
Theoretical length distribution of *k*-mismatch common substring extensions. The *expected* number of *k*-mismatch extensions of length *m* returned by *kmacs* was calculated using Eq. (17), distinguishing between ‘homologous’ and ‘background’ matches, for a pair of sequences of length $L=500$ kb with a match probability of $p=0.5$ for $k=10$ (top) and $k=70$ (bottom) for $20\le m \le 160$. A large enough value of *k* is necessary to detect the second peak in the distribution that corresponds to the ‘homologous’ matches

To further process the length distribution returned by the modified *kmacs*, we implemented a number of *Perl* scripts. First, the length distribution of the *k*-mismatch common substrings is smoothed using a window of length *w*. Next, we search for the second local maximum in this smoothed length distribution. This second peak should represent the *homologous* *k*-mismatch common substrings, while the first, larger peak represents the *background* matches, see Figs. 4 and 5. A simple script identifies the position $m^*$ of the second highest local peak under two side constraints: we require the height $N(m^*)$ of the second peak to be substantially smaller than the global maximum, and we require that $N(m^*)$ is larger than $N(m^*-x)$ for some suitable parameter *x*. Quite arbitrarily, we required the second peak to be 10 times smaller than the global maximum peak, and we used a value of $x=4$. These constraints were introduced to prevent the program to identify small side peaks within the background peak. Finally, we use the position $m^*$ of the second largest peak in the smoothed length distribution to estimate the match probability *p* in an alignment of the two input sequences using expression (19). The usual *Jukes-Cantor* correction is then used to estimate the number of substitutions per position that have occurred since the two sequences separated from their last common ancestor.

We should mention that our algorithm is not always able to output a distance value for two input sequences. It is possible that the algorithm fails to find a second maximum in the length distribution of the *k*-mismatch common substrings. This can happen, for example, for distantly related sequences where the ‘homologue’ and the ‘background’ peak are too close together such that the ‘homologous’ peak is obscured by the ‘background’ peak, see Fig. 5 for an example. In this case no distance can be calculated by our algorithm.

## Test results

**Fig. 6:**
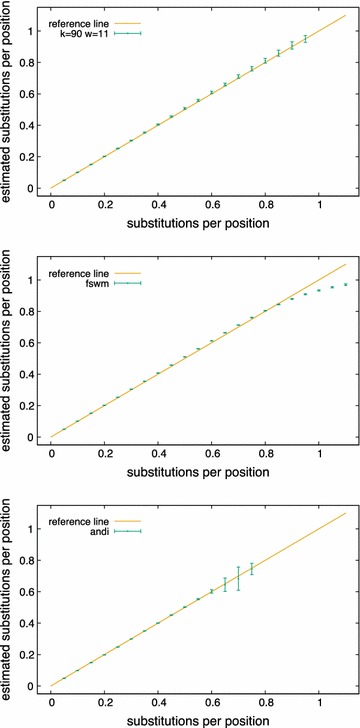
Estimated distances—i.e. estimated average number of substitutions per position—for simulated sequence pairs, plotted against the ‘real’ distances—i.e. substitution probabilities used in the simulations, for pairs of sequences of length $L=500$ kb. We applied our own approach with parameters $k=90$ and $w=31$ (top) as well as *Filtered Spaced Word Matches* (middle) and *andi* (bottom)

To evaluate our approach, we used simulated and real-world genome sequences. As a first set of test data, we generated pairs of simulated DNA sequences of with varying evolutionary distances and compared the distances estimated with our algorithm—i.e. the estimated number of substitutions per position—to their ‘real’ distances. For each distance value, we generated 100 pairs of sequences of length 500 kb each and calculated the average and standard deviation of the estimated distance values. Figure 6 shows the results of these test runs with a parameter $k=90$ and a smoothing window size of $w=31$, with error bars representing standard deviations. A program run on a pair of sequences of length 500 kb took less than a second.

Figure 4 shows the length distribution for one of these sequence pairs with various values for *w*. In Fig. 6, the results are reported for a given distance value, if distances could be computed for at least 75 out of the 100 sequence pairs (as mentioned above, it is possible that our program does not output a distance value since no second maximum could be found in the length distribution of the *k*-mismatch common substrings). As can be seen in the figure, our approach accurately estimates evolutionary distances up to around 0.9 substitutions per position. For larger distances, the program did not return a sufficient number of distance values, so no results are reported here. To demonstrate the influence of the parameter *k*, we plotted in Fig. 5, for a given set of parameters, the expected number of *k*-mismatch common substring extensions of length *m*, calculated with Eq. (17), using varying values of *k*.

**Fig. 7:**
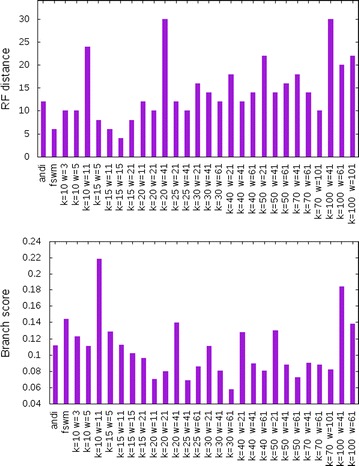
Evaluation of alignment-free methods for phylogeny reconstruction. Various methods were evaluated on on a set of 27 primate mitochondrial genomes. *Robinson-Foulds* distances (top) and *branch scores* (bottom) were calculated to measure the difference between the resulting trees and a reference tree obtained with *Clustal* $\Omega$ and *Neighbour Joining*

As a real-word test case, we used a set of 27 mitochondrial genomes from primates that has been used as benchmark data in previous studies on alignment-free sequence comparison. We applied our method with different values of *k* and with different window lengths *w* for the smoothing. In addition, we ran the programs *andi* [25] and our previously published program *Filtered Spaced-Word Matches (FSWM)* [29] on these data. As a reference tree, we used a tree calculated with *Clustal* $\Omega$ [31] and *Neighbour Joining* [32]. To compare the produced trees with this reference trees, we used the *Robinson-Foulds* distance [33] and the *branch score* distance [34] as implemented in the *PHYLIP* program package [35]. Figure 7 shows the performance of our approach with different parameter values and compares them to the results of *andi* and *FSWM*. For the parameter values shown in the figure, our program was able to calculate distances for all ${27 \atopwithdelims ()2}=351$ pairs of sequences. The total run time to calculate the 351 distance values for the 27 mitochondrial genomes was less than 6 s. Note that the time and memory consumption of our approach essentially depend on *kmacs*, the scripts that process the output of *kmacs* are negligible. For a discussion of the time and space complexity of our software, we therefore refer to our previous paper on *kmacs* [13].

## Discussion

In this paper, we introduced a new way of estimating phylogenetic distances between genomic sequences. We showed that the average number of substitutions per position since two sequences have separated from their last common ancestor can be accurately estimated from the position of local maximum in the smoothed length distribution of *k*-mismatch common substrings. To find this local maximum, we used a naive search procedure. Two parameter values have to be specified in our approach, the number *k* of mismatches and the size *w* of the smoothing window for the length distribution. Table 1 shows that our distance estimates are reasonably stable for a range of values of *k* and *w*.

**Table 1:** Distance values calculated with our algorithm for a pair of simulated sequences of length $L=500$ kb with a match rate of $p=0.5$, corresponding to a distance of 0.824 substitutions per position

	k = 30	k = 50	k = 70	k = 90	k = 120	k = 150	k = 200
w = 1	0.665	0.809	0.935	0.897	0.794	0.781	0.995
w = 5	–	0.839	0.835	0.784	0.783	0.773	0.880
w = 11	–	–	0.869	0.808	0.788	0.781	0.863
w = 21	–	–	0.813	0.824	0.824	0.804	0.817
w = 31	–	–	0.813	0.824	0.824	0.829	0.835
w = 51	–	–	–	–	0.824	0.819	0.820

Dashes indicate that no distance value could be calculated since our algorithm could not find the second local maximum in the smoothed length distribution of the *k*-mismatch common substrings

A suitable value of the parameter *k* is important to separate the ‘homologous’ peak from the ‘background’ peak in the length distribution of the *k*-mismatch common substrings. As follows from Theorem 2, the distance between these two peaks is proportional to *k*. The value of *k* must be large enough to ensure that the homologous peak has a sufficient distance to the background peak to be detectable, see Fig. 5. On the other hand, *k* should not be too large. All considerations in this paper are based on the assumtion that *k*-mismatch common substrings are either *homologue* or *background*, which is the case under our indel-free model of sequence evolution. For sequences with insertions and deletions, however, an un-gapped segment pair may contain both homologous *and* background regions, if it involves indels. If *k* is large, *k*-mismatch common substrings tend to be long, and ‘mixed’ *k*-mismatch common substrings, including both background and homologue segments, will distort our distance estimates. This seems to be the reason why in Fig. 7 our results deteriorate if *k* becomes too large. One possible solution to this problem would be to recognize ‘mixed’ *k*-mismatch common substrings by the distribution of their mismatches and to exclude them from the length statistics. This might allow us to increase *k* without running into the above mentioned problems, so one could achieve a better separation of ‘background’ and ‘homologous’ peaks. We are planning to investigate the effect of indels on our approach in a subsequent study.

Specifying a suitable size *w* of the smoothing window is also important to obtain accurate distance estimates; a large enough window is necessary to avoid ending up in a local maximum of the raw length distribution. For the data shown in Fig. 4, for example, our approach finds the second maximum of the length distribution at 179 if a window width of $w=31$ is chosen. From this value, the match probability *p* is estimated as

$$\begin{aligned} \hat{p} = \frac{179+1-90}{179+1} = 0.5 \end{aligned}$$

using Eq. (18), corresponding to 0.824 substitutions per position according to the *Jukes-Cantor* formula. This was exactly the value that we used to generate this pair of sequences. With window lengths of $w=21$ and $w=1$ (no smoothing at all), however, the second local maxima of the length distribution would be found at 181 and 171, respectively, leading to estimates of 0.808 ($w=11$) and 0.897 ($w=1$) substitutions per position. If the width *w* of the smoothing window is too large, on the other hand, the second peak may be obscured by the first ‘background’ peak. In this case, no peak is found and no distance can be calculated. In Fig. 4, for example, this happens with if a window width $w=51$ is used. Further studies are necessary to find out suitable values for *w* and *k*, depending on the length of the input sequences.

Finally, we should say that we used a rather naive way to identify possible homologies that are then extended to find *k*-mismatch common substrings. As becomes obvious from the size of the homologous and background peaks in our plots, our approach finds far more background matches than homologous matches. Reducing the noise of background matches should help to find the position of the homologous peak in the length distributions. We will therefore explore alternative ways to find possible homologies that can be used as starting points for *k*-mismatch common substrings.
